# Critical role of androgen receptor level in prostate cancer cell resistance to new generation antiandrogen enzalutamide

**DOI:** 10.18632/oncotarget.10926

**Published:** 2016-07-29

**Authors:** Julia Hoefer, Mohammady Akbor, Florian Handle, Philipp Ofer, Martin Puhr, Walther Parson, Zoran Culig, Helmut Klocker, Isabel Heidegger

**Affiliations:** ^1^ Department of Urology, Division of Experimental Urology, Medical University of Innsbruck, Austria; ^2^ School of Biosciences and Veterinary Medicine, University of Camerino, Camerino, Italy; ^3^ Institute of Legal Medicine, Medical University of Innsbruck, Innsbruck, Austria; ^4^ Forensic Science Program, The Pennsylvania State University, University Park, Pennsylvania, USA; ^5^ Center of Biomolecular and Cellular Engineering, International Clinical Research Center, St. Anne's Hospital, Brno, Czech Republic

**Keywords:** prostate cancer, enzalutamide resistance, androgen receptor, AR-V7, AR gene amplification

## Abstract

Enzalutamide is an androgen receptor (AR) inhibitor approved for therapy of metastatic castration resistant prostate cancer. However, clinical application revealed that 30 to 40% of patients acquire resistance after a short period of treatment. Currently, the molecular mechanisms underlying such resistances are not completely understood, partly due to a lack of model systems. In the present study we established three different cellular models of enzalutamide resistance including a cell line with wild type AR (LAPC4), DuCaP cells which overexpress wild-type AR, as well as a cell which has been adapted to long term androgen ablation (LNCaP Abl) and harbors the AR T878A mutation. After 10 months of cultivation, sustained growth in the presence of enzalutamide was achieved. When compared to controls, resistant cells exhibit significantly decreased sensitivity to enzalutamide as measured with ^3^[H]thymidine incorporation and WST assay. Moreover, these cell models exhibit partly re-activated AR signaling despite presence of enzalutamide. In addition, we show that enzalutamide resistant cells are insensitive to bicalutamide but retain considerable sensitivity to abiraterone. Mechanistically, enzalutamide resistance was accompanied by increased AR and AR-V7 mRNA and protein expression as well as AR gene amplification, while no additional AR mutations have been identified.

## INTRODUCTION

Enzalutamide (Xtandi®) is an oral androgen receptor (AR) inhibitor which has been developed for the treatment of prostate cancer (PCa). Enzalutamide blocks binding of androgens to AR, nuclear translocation of AR as well as AR-mediated DNA binding [1]. According to the literature, enzalutamide lacks partial agonistic activity which has been described for its predecessor bicalutamide [2]. After proving its efficacy in preclinical models [1], a large Phase III study (AFFIRM), in which overall survival increased from 13.6 to 18.4 months, demonstrated its benefit in the treatment of metastatic castration resistant prostate cancer (mCRPC) in patients post chemotherapy and the drug was finally approved in 2013 [3]. A follow-up study (PREVAIL) evaluating enzalutamide in mCRPC patients before chemotherapy confirmed increased overall survival in enzalutamide treated patients (from 30.2 to 32.4 months), leading to clinical registration also in the pre-chemotherapy setting [4].

Despite obvious benefits, application of the drug in a clinical setting revealed that not all patients respond to new generation AR modulators including enzalutamide [5]. Numerous patients show endogenous resistance to the drug. Moreover, a considerable proportion of patients develop resistance during treatment, leading to biochemical or symptomatic recurrence after a short period of regression. Thus, identification of biomarkers which allow distinguishing patients who are likely to respond to enzalutamide from those who will not benefit from the drug is urgently needed. In addition, it is essential to develop new therapy strategies and to address the challenging question of how currently available drugs should be ideally sequenced in order to prevent or overcome the occurrence of resistances. These aims require the availability of different cellular models of enzalutamide resistance which allow for detailed analysis of the underlying mechanisms. There is evidence that AR splice variants such as the AR splice variant 7 (ARV7) [6] or point mutations in the DNA regions encoding for the ligand binding domain of AR [7] are involved in enzalutamide resistance. However, until now the molecular mechanisms underlying an endogenous or acquired insensitivity to enzalutamide are not completely clarified, in part due to a lack of cellular models which are essential for detailed analysis of molecular changes that occur during enzalutamide therapy.

In the present study we aimed to uncover a potential involvement of AR expression in enzalutamide resistance. We established three different cellular models of enzalutamide resistance including a cell line with wild type AR (LAPC4), a cell line overexpressing wild-type AR (DuCaP) as well as a cell line (LNCaP Abl) which has been generated by long term androgen ablation from LNCaP, which harbor a mutated AR (T878A) [8, 9]. Using these cell lines, we investigated potential changes in the AR gene, mRNA and protein expression.

## RESULTS

### Generation of enzalutamide resistant cell lines

**Figure 1:**
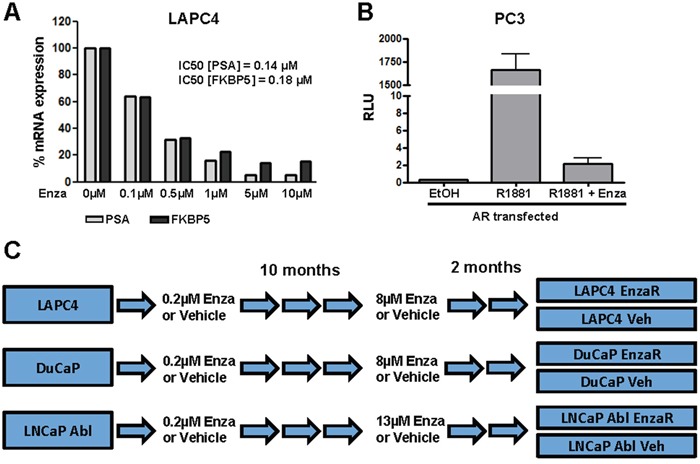
Generation of enzalutamide resistant cell lines **A.** LAPC4 cells were cultured in RPMI + 1 nM DHT and treated with increasing concentrations of enzalutamide for 48 h. mRNA expression of AR target genes PSA and FKBP5 was analyzed using RT-qPCR. **B.** AR reporter gene assay after treatment with R1881 [1 nM] or enzalutamide [10 μM] or both was performed with PC-3 cells ectopically expressing AR, androgen responsive elements and a TATA-box in framework of nanoluc reporter vector was measured by Nano-Glo Dual-Luciferase Assay and normalized to control luciferase activity **C.** Treatment scheme for the generation of enzalutamide resistant cell lines. LAPC4, DuCaP and LNCaP abl were cultured in the presence of increasing doses of enzalutamide or vehicle (EtOH) for 10 months (approximately 50 passages) until the final concentrations (8 μM for LAPC4 and DuCaP, 13 μM for LNCaP Abl) were reached. Cell lines were further cultured for 2 months in the presence of the final enzalutamide concentrations.

To define a starting concentration of enzalutamide for the generation of resistant cell lines, we treated LAPC4 cells with increasing concentrations of the drug (0.1 μM – 10 μM) and measured expression of AR target genes FKBP5 and PSA. As shown in Figure 1A, enzalutamide inhibited AR signaling dose dependently after 48 hours (IC50: 0.18 μM and 0.14 μM for PSA and FKBP5, respectively). Moreover, enzalutamide successfully inhibited reporter gene activity after treatment with the synthetic androgen R1881 (Figure 1B). On the basis of these results, we started to culture DuCaP, LAPC4 and LNCaP Abl parental cell lines in the presence of 0.2 μM enzalutamide or vehicle (EtOH) in order to generate enzalutamide resistant and vehicle control sub-cell lines, respectively. Enzalutamide concentration was incrementally increased up to a final concentration of 8 μM (DuCaP, LAPC4). Due to the fact that LNCaP abl cells are already adapted to growth in androgen ablated conditions, these cells were less sensitive and did not exhibit significant cell death or growth arrest up to a concentration of 10μM enzalutamide. We therefore increased the final concentration for LNCaP abl EnzaR to 13μM. After 10 months, control and resistant cells showed comparable growth behavior in the presence of the final concentrations of vehicle or enzalutamide, respectively. To ensure sustained survival and growth, cells were kept in these conditions for another 2 months. A schematic overview of the procedure is presented in Figure 1C.

### Newly generated sub-cell lines show decreased sensitivity to enzalutamide

**Figure 2:**
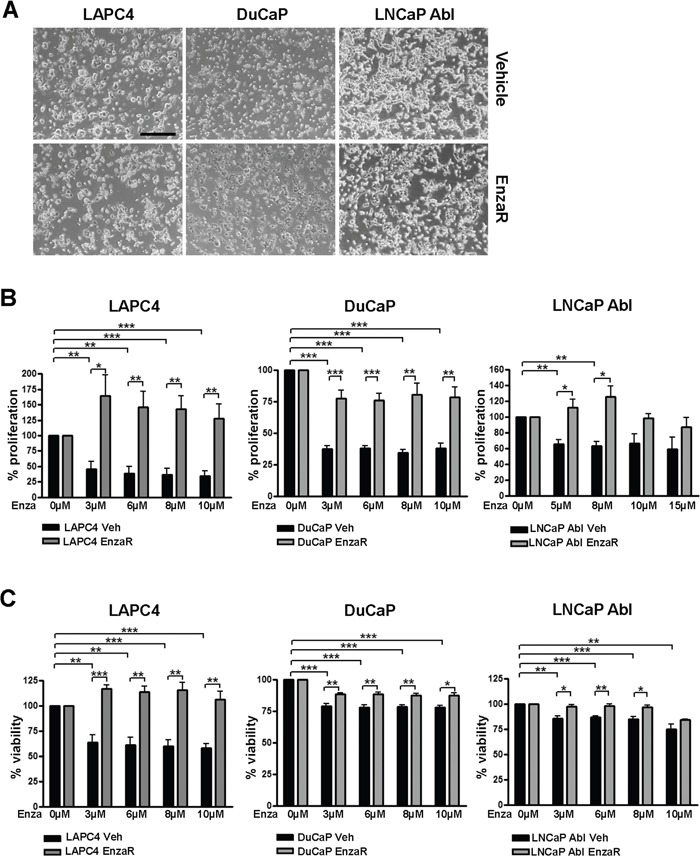
Enzalutamide resistant cell lines exhibit decreased sensitivity to enzalutamide **A.** Representative images of vehicle and EnzaR cell lines were taken after 12 months of culturing. 4x objective. Scalebar: 500μm. **B, C.** Determination of the degree of enzalutamide resistance. Proliferation (B) and viability (C) in DuCaP, LAPC4 and LNCaP Abl vehicle and enzalutamide resistant cell lines after 6 days of exposure to different concentrations of enzalutamide were measured by ^3^[H]thymidine incorporation assay and WST assay, respectively. Ethanol was used as control. Data represent mean +SEM from at least 3 independent experiments. *;p=<0.05. **;p=<0.01. ***;p=<0.001

After one year of cultivation, enzalutamide resistant (EnzaR) and vehicle treated (Veh) cell lines exhibit a comparable morphology (Figure 2A). In order to evaluate the degree of resistance of long term enzalutamide-treated cell lines, we measured viability using WST assay as well as proliferation by ^[3]^H thymidine incorporation assay following treatment with different doses of vehicle or enzalutamide. Given the expected insensitivity of the EnzaR sub cell-lines, enzalutamide doses above the starting concentration of the selection process were selected (3-10 μM for LAPC4, DuCaP or 5-15 μM for LNCaP Abl). As expected, in all three control (vehicle) cell lines we observed maximal inhibition of proliferation (Figure 2B) and viability (Figure 2C) already after treatment with the lowest enzalutamide concentration. With respect to viability, LAPC4 exhibited higher sensitivity to enzalutamide than DuCaP or LNCaP Abl cells. Notably, all three enzalutamide resistant sub-lines exhibit significantly decreased sensitivity to the drug up to 10 μM. Of note, in the resistant LAPC4 (LAPC4 EnzaR) sub-line we observed even a significant increase in proliferation and viability in the presence of enzalutamide, when compared to vehicle treated controls (Figure 2B and 2C). Measurement of the expression of AR target genes FKBP5, KLK2 and PSA revealed that resistant sub cell-lines regain active AR signaling despite presence of enzalutamide, although target gene expression patterns revealed some cell type and gene specific heterogeneity (Supplementary Figure S1). Of the three target genes, FKBP5 was not upregulated in LAPC4 cells, neither was KLK2 in LNCaP abl cells.

### Enzalutamide resistant sub-cell lines are partly sensitive to abiraterone but insensitive to bicalutamide

**Figure 3:**
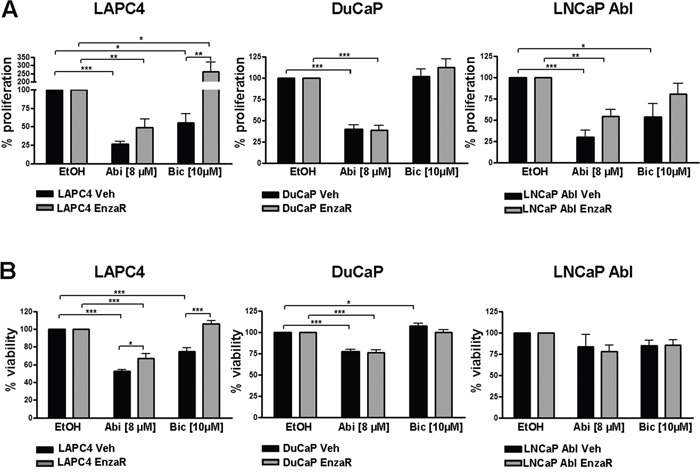
Determination of possible cross resistances of enzalutamide resistant cell lines with abiraterone or bicalutamide Proliferation **A.** and viability **B.** in DuCaP, LAPC4 and LNCaP Abl vehicle and enzalutamide resistant cell lines after 6 days of exposure to 8 μM abiraterone or 10 μM bicalutamide was measured by ^3^[H]thymidine incorporation assay and WST assay, respectively. Ethanol was used as control. Data represent mean +SEM from at least 3 independent experiments. *;p=<0.05. **;p=<0.01. ***;p=<0.001

Next, we aimed to evaluate possible cross-resistances of the newly generated enzalutamide resistant cell lines with other drugs that target the AR pathway, such as bicalutamide or abiraterone. Briefly, bicalutamide is an older, non-steroidal anti-androgen which is clinically still used as an adjuvant agent in patients receiving radiation therapy due to intermediate or high risk PCa. Moreover, bicalutamide prevents the flare up phenomena in LHRH agonist therapy [10]. Abiraterone on the other hand, blocks biosynthesis of androgens by inhibition of CYP17A1, however, it has been suggested that it may in addition act as direct AR antagonist [11]. As enzalutamide, also abiraterone is approved for the treatment of mCRPC before or after chemotherapy. Interestingly, cross resistance of enzalutamide resistant PC346C cells to abiraterone has been reported in 2013 [12]. Thus, we assessed the degree of sensitivity of the newly generated enzalutamide resistant DuCaP, LAPC4 and LNCaP Abl sub-cell lines to abiraterone and bicalutamide. Our data clearly show that abiraterone is still significantly decreasing proliferation in all three and viability in two of three sub-lines, although the growth-inhibitory effect of abiraterone was reduced in LAPC4 and LNCaP Abl EnzaR compared to vehicle controls (Figure 3A and 3B). These findings indicate a potential therapeutic benefit of applying abiraterone to patients who relapsed after enzalutamide.

While we observed complete resistance to bicalutamide in both DuCaP sub-cell lines, LAPC4 and LNCaP Abl control cell lines are responsive to the drug. Notably, we detected decreased bicalutamide sensitivity in LNCaP Abl EnzaR compared to controls while LAPC4 EnzaR not only lost their sensitivity to bicalutamide, but exhibited even enhanced proliferation in the presence of the drug (Figure 3A). Measurement of viability under the same conditions yielded similar results (Figure 3B).

### Development of enzalutamide resistance is not caused by a gain of AR mutations

In order to uncover the mechanisms leading to enzalutamide resistance, we first hypothesized that mutations in the AR gene may lead to decreased sensitivity to the drug. However, sequencing of AR coding cDNA revealed that LAPC4 and DuCaP EnzaR sub-lines did not gain any AR mutations compared to the vehicle treated controls. Similarly, LNCaP Abl EnzaR did not harbor additional AR mutations other than the known LNCaP mutation T878A [8, 9].

### Enzalutamide resistance is accompanied by increased expression of full length AR and AR variant 7

**Figure 4:**
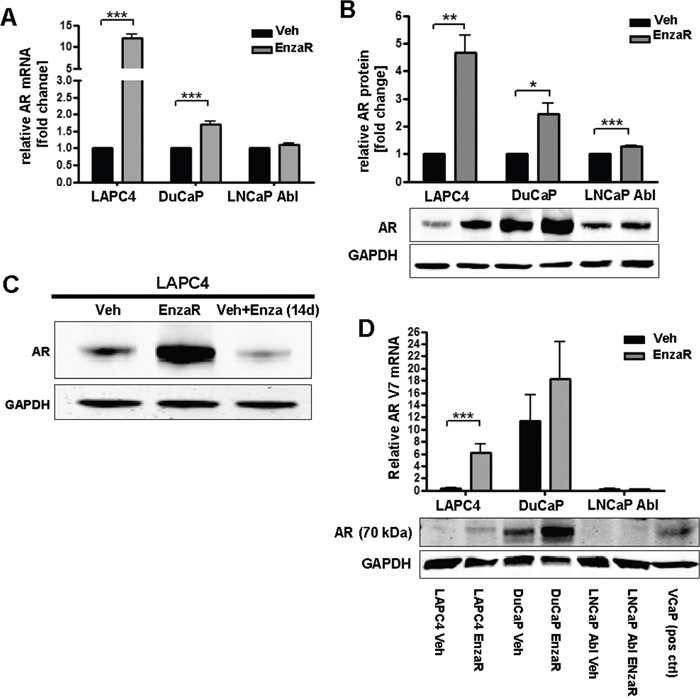
Enzalutamide resistant cell lines exhibit increased AR expression **A.** AR mRNA expression was assessed by qRT-PCR. Data represent mean +SEM from 4 independent experiments. *;p=<0.05. **;p=<0.01. ***;p=<0.001. **B.** Statistical analyses and representative Western blot images of full length AR protein expression. Data represent mean +SEM from 3 independent experiments. *;p=<0.05. **;p=<0.01. ***;p=<0.001. **C.** Western blot of LAPC4 Veh and LAPC4 EnzaR, as well as in LAPC4 vehicle cells which were treated for 2 weeks with enzalutamide [8 μM]. **D.** Upper panel: Statistical analysis of AR-V7 mRNA expression. Data represent mean +SEM from 4 independent experiments. *;p=<0.05. **;p=<0.01. ***;p=<0.001. Lower panel:Representative Western blot image of AR variant observed at 70 kd size (V7). First lane represents Marker band the 75 kDa size. Last lane represents VCaP lysate as positive control for V7 expression.

Since we did not identify additional AR mutations which account for development of enzalutamide resistance, we next assessed AR expression level in vehicle or EnzaR sub-cell lines. AR mRNA significantly increased in LAPC4 and DuCaP EnzaR cells, while in LNCaP Abl EnzaR we did not observe major changes in AR mRNA expression (Figure 4A). Similarly, AR full length protein was highly elevated in enzalutamide resistant LAPC4 and DuCaP cells. LNCaP Abl EnzaR exhibit only a slight increase in full length AR expression (Figure 4B). Short term treatment (14 days) of LAPC4 vehicle cells with 8μM enzalutamide (Figure 4C) clearly demonstrated that AR overexpression is not a short term effect of drug treatment but develops as a long term adaptation during acquisition of resistance. It has been suggested that presence of a truncated AR variant (AR-V7) is associated with resistance to enzalutamide [13]. Thus, we assessed AR-V7 mRNA and protein levels in the newly generated EnzaR sub cell-lines. As shown in Figure 4D, LAPC4 cells which *per se* do not exhibit relevant levels of V7 mRNA or protein, acquired V7 mRNA and protein expression with development of enzalutamide resistance. In DuCaP on the other hand, V7 was present even in the control cell line and further increased in DuCaP EnzaR. In contrast, neither LNCaP Abl vehicle nor EnzaR exhibited detectable amounts of truncated AR variants (Figure 4D).

**Figure 5:**
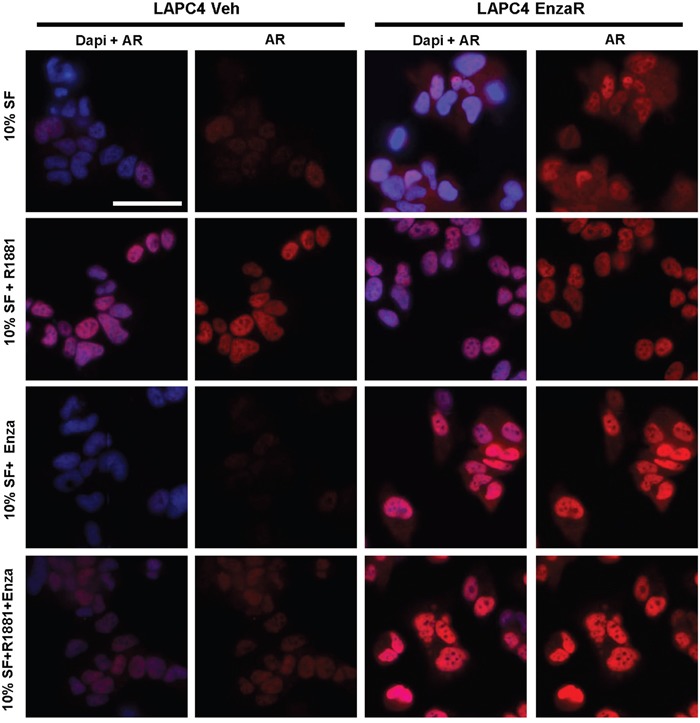
Immunofluorescence staining of vehicle or enzalutamide resistant LAPC4 cells Cells were cultured in medium containing 10% charcoal stripped FBS (SF), supplemented with vehicle (EtOH), 1 nM R1881, or 8μM enzalutamide as indicated. AR was detected using mouse anti AR (Biogenex) and visualized using AlexaFluor 488 donkey anti mouse secondary antibody. Nuclei were counterstained with DAPI. Magnification: 40x. Scalebar: 50μm.

Changes in AR expression in enzalutamide resistant cells were further confirmed by immunofluorescence (Figure 5). In the LAPC4 vehicle cells, AR staining was weak under serum starvation conditions (10% SF) and increased after R1881 treatment. As expected, enzalutamide inhibited basal expression as well as R1881 driven AR upregulation. In LAPC4 EnzaR on the other hand, AR was elevated already under serum starvation and did not significantly change upon R1881 addition. Notably, presence of enzalutamide further increased nuclear AR, both in the absence and presence of R1881 (Figure 5). A similar situation was found in DuCaP cell lines (Supplementary Figure S2).

### AR gene amplification is one mechanism of increased AR expression in enzalutamide resistant cells

**Figure 6:**
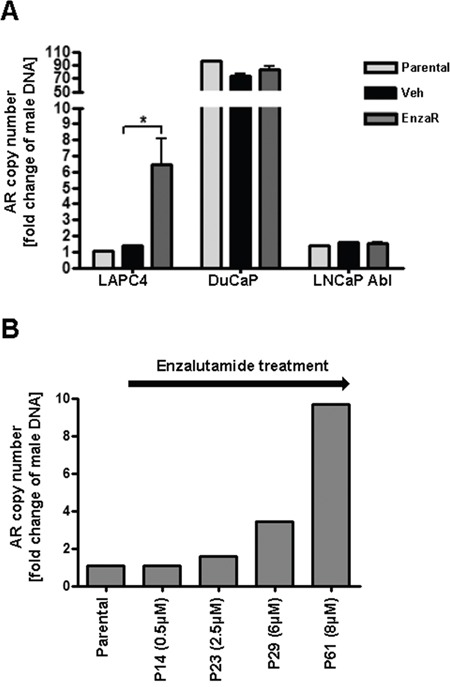
Enzalutamide resistant LAPC4 acquired AR gene amplification **A.** AR gene amplification was determined by duplex qPCR of genomic DNA amplifying an AR Exon 1 fragment (Chr Xq12) in relation a POLG Exon 3 fragment (Chr 15q25). AR/POLG copy number ratios were calculated as fold change of normal male DNA which harbors 1 copy of AR. Data represent mean +SEM from 3 independent PCR analyses. *;p=<0.05 **B.** Analysis of AR gene amplification over time during generation of enzalutamide resistant LAPC4 cells starting from parental LAPC4. P = Passage.

In order to further uncover the molecular background underlying increased AR expression in enzalutamide resistant cells, we investigated AR gene copy number in all established vehicle or EnzaR sub-cell lines. As an additional control, we included corresponding parental cells which had been frozen before long term treatments were started. AR gene amplification was determined by duplex qPCR of genomic DNA amplifying an AR Exon 1 fragment (Chr Xq12) in relation to a POLG Exon 3 fragment (Chr 15q25). AR/POLG copy number ratios were calculated as fold change of normal male DNA which harbors 1 copy of AR. As expected, parental as well as vehicle treated LAPC4 cells exhibit a normal AR copy number. Strikingly, we detected a ~8-fold amplification of AR gene in enzalutamide resistant LAPC4 (Figure 6A) which was gained gradually over time during passaging with increasing doses of enzalutamide (Figure 6B). Parental and vehicle-treated DuCaP cells on the other hand already exhibited a dramatic amplification of the AR locus (~ 80 copies) which was not further changed after long term treatment with the drug. Similarly, enzalutamide treatment did not influence the normal AR copy number in LNCaP Abl cells (Figure 6A).

## DISCUSSION

The AR is one of the major drivers of PCa progression and various changes in AR status such as AR amplifications or mutations lead to persistence of AR signaling in CRPC [14]. Therefore, numerous therapeutic approaches for advanced or castration resistant PCa target the androgen – AR signaling cascade. The new generation AR inhibitor enzalutamide has been shown to increase overall survival and suppress disease related symptoms when administered to mCRPC patients before or after chemotherapy. Despite of this, many patients do not respond to enzalutamide therapy or acquire resistance during treatment. It has been proposed that AR mutations or changes in AR expression might contribute to such insensitivities. However, until now the underlying mechanisms are not completely uncovered, in part due to a lack of appropriate models which would allow for detailed analysis. In the present study, we describe the cultivation and characterization of three enzalutamide resistant cell lines with different AR statuses. LAPC4 cells represent metastatic PCa cells with moderate levels of wild type AR. Moreover, we included LNCaP Abl cells, a LNCaP sub-line which has been grown in the absence of androgens for 60 passages [15]. These cells are androgen independent but -sensitive, thus representing a CRPC model [15]. LNCaP abl as well as the parental LNCaP harbor the T878A (formerly T877) AR mutation [16] which lies in the LBD of AR and is the most frequently reported substitution in prostate cancer [17]. DuCaP on the other hand, overexpress full length AR due to gene amplification as shown by us and others [18] and are in addition positive for AR-V7 splice variant, a variant which is frequently detected in patients resistant to abiraterone and enzalutamide therapy [19]. The newly generated enzalutamide resistant cell lines thus represent useful models for analysis of resistance mechanisms in cells with distinct AR statuses, reflecting heterogeneity in PCa patients.

Reviewing the literature, several possible mechanisms of enzalutamide resistance are discussed including AR splice variants, mutations or amplifications. One study showed that enzalutamide treatment of LNCaP causes selection of a F876L missense mutation in the AR ligand binding domain which leads to partial agonism and thus resistance to the drug [7]. However, in our study development of enzalutamide resistance in the used cell lines was not accompanied by the appearance of new AR mutations. This is not surprising, considering that the overall frequency of somatic AR mutations in CRPC patients is less than 10% [20, 21]. Moreover, the experimental procedure to generate enzalutamide resistant LNCaP in the study mentioned above [7] was different from our protocol. In the present study, we were able to show significantly increased AR mRNA and protein levels in 2 out of 3 enzalutamide resistant sub-lines, suggesting AR overexpression as a common mechanism for enzalutamide resistance. This is in line with findings from Yamamoto et al. who detected high levels of full-length AR and AR variants in enzalutamide resistant LNCaP [22]. However, the underlying mechanisms causing AR overexpression seem to be diverse among different cell lines. In LAPC4 cells which have a normal AR copy number and express moderate levels of wild type AR, long term enzalutamide treatment results in step-wise AR gene amplification and consequently in increased AR mRNA (10-fold) and protein (5-fold) levels. In line with this, it has been reported that AR amplifications are rare in untreated tumors but occur at a higher frequency in cases of endocrine therapy failure [23] indicating an important role for AR gene amplification in hormone therapy response. Indeed, one study suggested that AR amplification in patients after enzalutamide treatment correlates with resistance to the drug [24] and a very recent report showed that AR copy number gain occurs in 36% of patients after enzalutamide therapy and is associated with decreased progression free and overall survival [25]. In the present study we show that DuCaP exhibit a ~60-fold AR gene amplification which results in high AR protein levels already in the parental cell line. Although no further amplification of the AR gene was present in DuCaP EnzaR, we detected a significant increase in both, AR mRNA (3-fold) and protein (2.5-fold), which is hence caused by other mechanisms than gene amplification. In this context it has to be stated that AR is upregulated in most cases of CRPC but only in one third of cases this could be attributed to AR gene amplification [23]. These findings clearly demonstrate involvement of other mechanisms of AR upregulation, such as enhanced AR transcription or stabilization [26].

Recently, it has been shown that presence of AR splice variant 7 (ARV7) in bone marrow biopsies from patients under enzalutamide therapy was associated with insensitivity to the drug [27]. In addition, Antonarakis et al. found lower response rates and shorter progression free survival in enzalutamide treated patients with detectable amounts of V7 in circulating tumor cells [13]. In line with these findings, we were able to confirm increased expression of a 70 kDa AR variant in 2 out of 3 enzalutamide resistant sub-lines. Moreover, our data prove that V7 is expressed at low levels already in parental DuCaP cells. We hypothesize that presence of V7 in this cell line, together with amplification of the AR gene, may explain the observed general insensitivity of DuCaP to bicalutamide, and a lower sensitivity to enzalutamide compared to LAPC4. Taken together, these results strongly suggest a role for AR-V7 in the development of enzalutamide resistance. Thus, it might be worthwhile to therapeutically inhibit AR variant expression in order to overcome enzalutamide resistance. In this context, one study already showed that the antihelminthic drug niclosamide is able to potently block AR-V7 expression and consequently PCa cell growth in enzalutamide sensitive and resistant cells [28].

Given that in enzalutamide resistant LNCaP Abl cells we did not detect relevant changes in AR full length or V7 levels, we conclude that also other molecules apart from the AR may be involved in enzalutamide resistance. In this context, it has been shown that enzalutamide induces autophagy in CRPC cell lines by AMPK activation and mTOR suppression [29]. In addition, it has been demonstrated that constitutively active STAT3 is accompanied with resistance to enzalutamide [30]. Most interestingly, Arora et al. showed that induction of glucocorticoid receptor (GR) expression is frequently found in enzalutamide resistant tumors and propose that in the presence of enzalutamide the GR may substitute for AR, by activating a certain subset of AR target genes, thereby promoting PCa progression [31]. Indeed, we show here that in LNCaP Abl EnzaR cells, KLK2 mRNA expression is dramatically decreased while no change in PSA and FKBP5 was detected (Supplementary Figure S1). Thus, future studies might assess the role of GR in LNCaP Abl EnzaR cells.

Van Soest et al. showed that enzalutamide resistant PC346C cells exhibit an almost complete cross resistance to abiraterone [12] which may be a cell line specific observation. Here, we demonstrate that enzalutamide resistant LAPC4, DuCaP and LNCaP Abl are partly sensitive to 8 μM abiraterone even though the degree of sensitivity was diminished in enzalutamide resistant LAPC4 and LNCaP Abl when compared to control cells. Of note, parental and enzalutamide resistant DuCaP exhibit comparable sensitivity to abiraterone, despite presence of AR-V7. This result may be explained by a study from Pfeiffer et al. who described an intracrine androgen synthesis in DuCaP cells which is further increased upon long term androgen depletion [32], indicating that both parental and long-term enzalutamide treated DuCaP cells rely at least to a certain degree on steroidogenesis, which renders them sensitive to abiraterone treatment. In summary, findings from us and others indicate that abiraterone might be beneficial at least in a subset of patients relapsing after enzalutamide treatment. In line with this hypothesis, there are two small clinical studies showing that abiraterone still exhibits modest overall activity in mCRPC patients who relapsed after enzalutamide, with large heterogeneity in the degree of response among different patients. Loriot et al. observed a ≥50% or ≥30% PSA drop upon abiraterone in 8 % or 18% of patients who previously relapsed under enzalutamide treatment [33]. In a second study, Noonan et al. found ≥50% or ≥30% PSA decline upon abiraterone in 3 % or 11% of patients pretreated with enzalutamide, respectively [34]. These studies and our own results underline the heterogeneity of prostate tumors regarding AR signaling addiction and resistance mechanisms and suggest that a subset of patients may benefit from abiraterone after enzalutamide failure. Considering the fact that for patients with metastatic CRPC therapy sequence still remains speculative with great differences in outcome (reviewed in [35]), it will be of importance to verify these clinical findings in larger patient cohorts with such a treatment sequence. Moreover, it has to be noted that in this study we only tested effects of a single abiraterone concentration (8 μM) at a single time point in the EnzaR sub-lines. Detailed analyses have to assess dose-response curves at different time points for abiraterone in those cell lines and the time course of development of possible abiraterone cross resistance.

Interestingly, enzalutamide resistant LAPC4 cells proliferate less under control conditions than under enzalutamide treatment. Moreover, enzalutamide resistant LAPC4 exhibit higher levels of nuclear AR in the presence of enzalutamide than without the drug. This observation may point to a switch from antagonistic to agonistic action of enzalutamide as described already for bicalutamide and may explain an enzalutamide withdrawal syndrome which was evident in a subset of patients [36]. In line with this hypothesis, Korpal et al. described an antagonist-to-agonist switch in enzalutamide resistant LNCaP due to a F876L mutation of AR [7]. However, we found no AR mutation in our enzalutamide resistant LAPC4 cells. Future studies have to carefully investigate this issue.

### Conclusion

In the present study we show for the first time the successful establishment of three different enzalutamide resistant cell lines, which provide an important tool for investigation of resistance mechanisms in cells with different AR statuses. We found that in these models enzalutamide resistance is not accompanied by AR mutations, while increased expression of full length and truncated AR as well as AR gene amplification might be involved in development of resistance. Furthermore, our results suggest that abiraterone treatment might be feasible in a subset of patients resistant to enzalutamide.

## MATERIAL AND METHODS

### Cell culture and chemicals

The human prostate cell lines LAPC4 (lymph node metastasis) were a gift from Dr. A. Cato (University of Karlsruhe, Germany). DuCaP (brain metastasis) were a gift from from Dr. J Schalken (Radboud University, Nijmegen, The Netherlands). The subline LNCaP Abl was previously established by our group after long term cultivation of LNCaP (lymph node metastasis) in steroid free medium as described earlier [15]. The identity of the used cell lines was confirmed by short tandem repeat analysis. LAPC4 and DuCaP cells were cultured in RPMI 1640 containing 10% fetal bovine serum (FBS; PAA Laboratories) and 2 mM glutamax (Thermo Fisher Scientific). LAPC4 were in addition supplemented with 1 nM dihydrotestosterone (DHT). LNCaP Abl cells were grown in RPMI 1640 containing 10% charcoal stripped FBS (HyClone, GE Healthcare) and 2 mM glutamax. Androgen stimulation experiments were performed using 1 nM of the synthetic androgen R1881 (Organon) dissolved in EtOH. Enzalutamide and abiraterone (both MedChemExpress) were dissolved in EtOH at a concentration of 1mM. Bicalutamide (AstraZeneca) was dissolved in DMSO at a concentration of 5mM.

### Generation of resistant cell lines

Cell lines were cultured in the presence of increasing doses of enzalutamide or vehicle (EtOH). Drug treatment was started using 0.2μM enzalutamide. Drug-containing medium was changed every third day. Cells were splitted when 80% confluency was reached. Enzalutamide concentration was increased when cells started to regrow in the presence of the drug at a growth rate similar to that of control cells until final concentrations (DuCaP, LAPC4: 8 μM; LNCaP Abl: 13 μM) were reached. Cells were then cultured for another 8 weeks in these conditions.

### Quantitative real time PCR (qRT-PCR)

Total RNA was isolated using the RNeasy mini kit (Qiagen, Hilden, Germany) and cDNA synthesis was performed using iScript Select cDNA synthesis kit (Biorad). Taqman qRT-PCR was performed as described elsewhere [37]. Expression was normalized to the endogenous reference TATA-Box binding protein (TBP) (forward 5-CACGAACCACGGCACTGATT-3; reverse 5-TTTTCTGCTGCCAGTCTGGAC-3; probe 5-FAM-TCTTCACTCTTGGCTCCTGTGCACA-TAMRA-3). The following primers and probes were used: AR (Fwd: 5′-AGGATGCTCTACTTCGCCCC-3′; Rev: 5′-ACTGGCTGTACATCCGGGAC-3′; Probe: 5′-FAM-TGGTTTTCAATGAGTACCGCATGCACA-TAMRA-3′), PSA (Fwd: 5′-GTCTGCGGCGGTGTTCTG-3′; Rev: 5′-TGCCGACCCAGCAAGATC-3′; Probe: 5′-FAM-CACAGCTGCCCACTGCATCAGGA-TAMRA-3′). For FKBP5 (Hs01561006_m1) and KLK2 (Hs00428383_m1), a taqman gene expression assay (Applied Biosystems) was used according to the manufacturer's protocol. Detection of AR-V7 was performed by SYBR green real time PCR employing the Power Sybr green PCR master mix (Applied Biosystems). The following primer sequences were used at a final concentration of 400nM each: V7 forward: 5′-CGGAAATGTTATGAAGCAGGGATGA-3′, V7 reverse: 5′- CTGGTCATTTTGAGATGCTTGCAAT – 3′ [38]. Efficiency of primers was calculated as 108% using serial dilutions of a plasmid carrying AR-V7.

### Western blot

Western blot was performed as previously described [37]. The following antibodies were used: anti-GAPDH (1:100000; Chemicon), anti-AR (N20; 1:500; Santa Cruz). As a positive control for truncated AR variant 7 band at 70 kDa size, a VCaP cell extract was included.

### Immunofluorescence

Immunofluorescence was performed as described previously [39]. Briefly, cells were seeded in standard growth medium in 6 well plates containing glass coverslips. On the next day, medium was changed to medium supplemented with 10% charcoal stripped FBS (SF) including 1 nM R1881, 8μM enzalutamide or both, as indicated. After 24h, cells were fixed with 4% paraformaldehyde and permeabilized with PBS / 1% BSA / 0.2% Triton X100. After a blocking step, cells were incubated with mouse anti-AR (Biogenex; 1:50) for 1 hour and subsequently probed with the labeled secondary antibody donkey anti mouse 555 (Invitrogen) for 1 hour. Cells were embedded using Vectashield mounting medium containing Dapi (Vector Laboratories). The cells were visualized using fluorescent microscopy on a Zeiss Axio Imager Z2 microscope.

### [^3^H]thymidine incorporation assay

Cells were seeded in triplicates onto 96-well plates. On the next day, cells were treated with enzalutamide, abiraterone, bicalutamide or vehicle (EtOH) as indicated. On day 5, 25 μL/well of diluted [^3^H]thymidine (1 μCi/well) were added for 24 h. DNA was harvested on 96-well filter plates (Perkin-Elmer). Scintillation fluid (50 μL) was added and radioactivity was quantified using a Chameleon 5025 liquid scintillation counter (HVD Life Sciences).

### Viability assay

Viability was assessed using WST reagent (Roche) according to the manufacturer's protocol.

### Reporter gene assay

AR reporter gene assay was performed with AR-negative PC-3 cells in 96-well plate format employing a synthetic promoter composed of two androgen responsive elements and a TATA-box ((ARE)_2_TATA) in framework of nanoluc reporter vector pNL1.1 [Nluc] (Promega). A PGK-promoter driven luciferase control vector (pGL4.53, Promega) was included for normalization. Per well, 30 ng of each, reporter and normalization control vector and 4 ng of AR-wild-type expression vector pSG5AR were mixed and complexed for 15 min with 0.2 μL of transfection reagent (Viafect, Promega) in 3 μL of transfection buffer and thereafter mixed with 50 μL of PC3 suspended in medium supplemented with 3% charcoal-stripped FCS (2×10^5^ cells/mL). After an incubation for 30 min at room temperature cell suspension was transferred to the wells, R1881 (1 nM), enzalutamide (10 μM) and vehicle were added and cells were incubated for 36 h before NanoLuc and firefly luciferase activities were measured using Chameleon 5025 instrument (HVD Life Sciences) and Nano-Glo Dual-Luciferase Assay (Promega) according to manufacturer's recommendations. Quadruplicate samples were measured for each treatment and nanoluc reporter gene activity was normalized to control luciferase activity.

### Sequencing of AR coding sequence

cDNA, prepared as described above, was used to PCR-amplify 3 overlapping AR cDNA fragments covering the entire AR coding sequence [40, 41]. cDNA fragments were purified using QIAquick PCR amplification kit (Qiagen) and sent for Sanger sequencing with appropriate sequencing primers (Microsynth) as described previously [40, 41]. Sequences were aligned to NCBI AR Reference Sequence NM_000044.3 using APE-A plasmid editor (v2.0.45) and checked for mutations.

### Measurement of gene amplification

Amplification of AR gene in parental, vehicle and EnzaR cell lines was assessed by duplex qPCR quantifiying AR (location Xq12, NCBI gene ID 367) in relation to POLG gene (polymerase gamma, location 15q25, NCBI gene ID 5428) in genomic DNA. Genomic DNA was isolated using QIAquick DNA isolation kit (Qiagen) and a duplex qPCR was performed with 20 ng of DNA using primer/probe sets AR_fw 5′-GACCTTAAAGACATCCTGAG-3′, AR_rev 5′CCCTAAGTAATTGTCCTTGG-3′, AR_probe FAM-5′-CAACTCCTTCAGCAACAGCAGC-3′_BHQ1 and POLG_fw 5′-AGAGCGTTACTCTTGGAC-3′, POLG_rev 5′-TCGGTCAAAGGAAACATTG-3′, POLG_probe YYE-5′-CACCACTAACTGCTCCTGCCA-3′BHQ1 on ABI 7500 FAST real-time PCR instrument (Life Technologies). Primers and probe were used at a concentration of 800 and 100 nM, respectively. PCR efficiency was calculated as 105%. The ratio AR/POLG DNA was calculated using the formula 2^−Δct^ and normalized to male control DNA, harboring 1 copy of AR gene.

## SUPPLEMENTARY FIGURES


