# Supplementary material for: Critical role of androgen receptor level in prostate cancer cell resistance to new generation antiandrogen enzalutamide
Source: Oncotarget. 2016 Jul 29;7(37):59781–94. doi: 10.18632/oncotarget.10926 (PMC5312348; doi:10.18632/oncotarget.10926)
Supplement: Supplementary file 1 [file oncotarget-07-59781-s001.pdf]

Critical role of androgen receptor level in prostate cancer cell resistance to new generation antiandrogen enzalutamide

SUPPLEMENTARY FIGURES

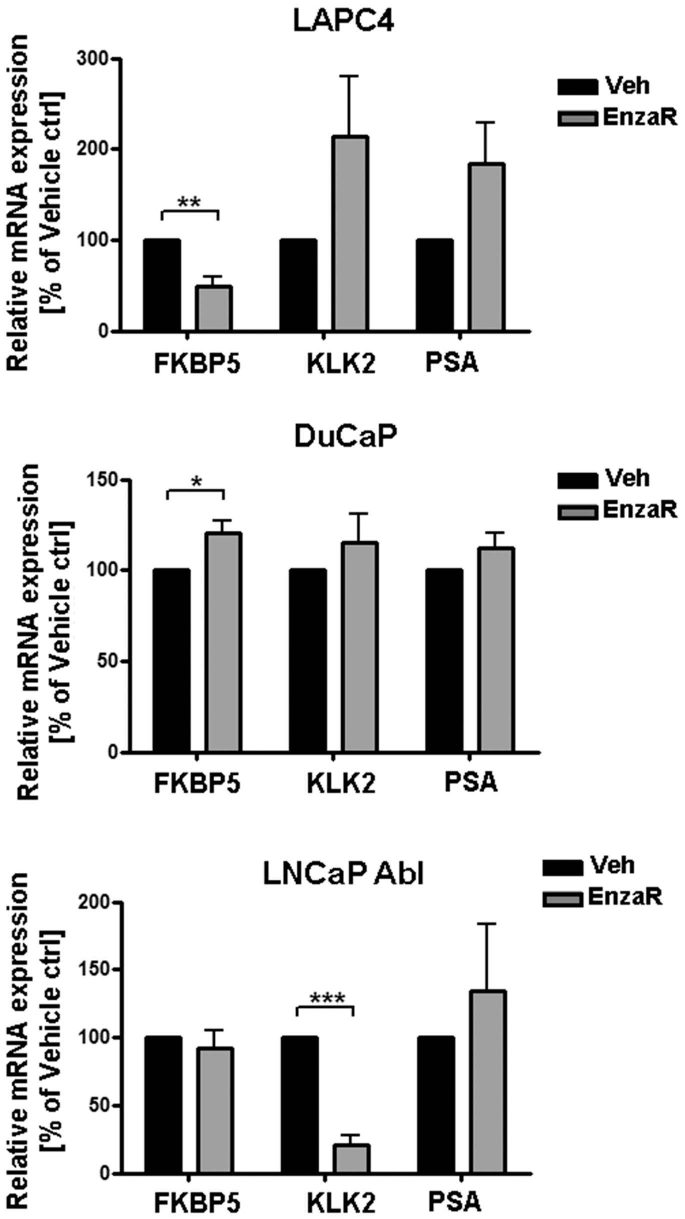

Supplementary Figure S1: Transcriptional activity of AR in vehicle and resistant cell lines was assessed using qPCR of AR target genes FKBP5, KLK2 and PSA. Cells were grown in standard culture media including the respective concentration of EtOH (vehicle) or enzalutamide, respectively. Data represent mean +SEM from 4 independent experiments. *;p=<0.05. **;p=<0.01. ***;p=<0.001.

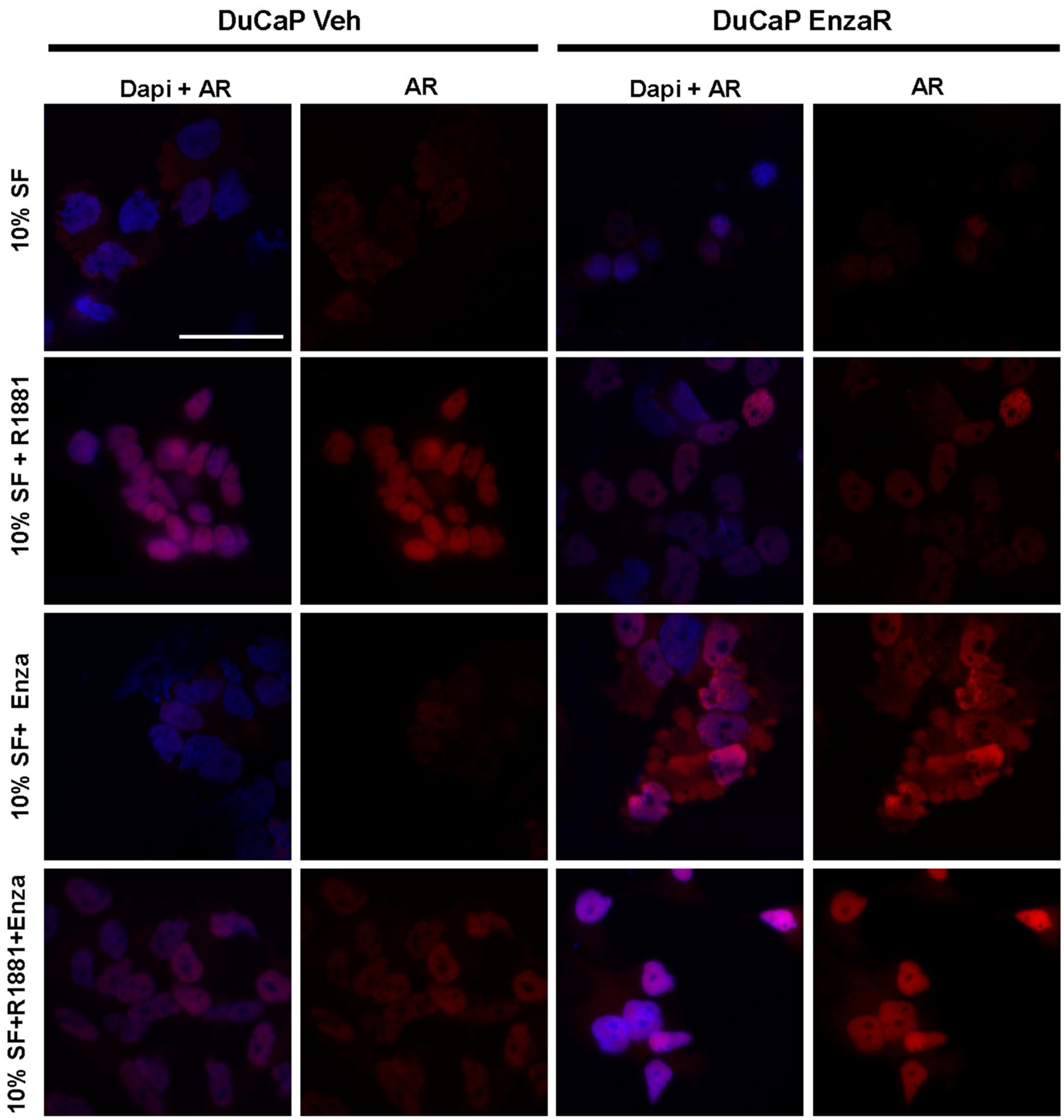

Supplementary Figure S2: DUCaP sub-lines were cultured in medium containing 10% charcoal stripped FCS (SF), supplemented with vehicle (EtOH), R1881, or enzalutamide as indicated. AR was detected using mouse anti AR (Biogenex) and visualized using AlexaFluor 488 donkey anti mouse secondary antibody. Nuclei were counterstained with DAPI. Magnification: 40x. Scalebar: 50µM.
